# Impact of a long-term tobacco-free policy at a comprehensive cancer center: a series of cross-sectional surveys

**DOI:** 10.1186/1471-2458-14-1228

**Published:** 2014-11-27

**Authors:** Cristina Martínez, Marcela Fu, Jose María Martínez-Sánchez, Laura Antón, Paz Fernández, Montse Ballbè, Ana Andrés, Anna Riccobene, Xisca Sureda, Albert Gallart, Esteve Fernández

**Affiliations:** Tobacco Control Unit, Cancer Control and Prevention Programme, Institut Català d’Oncologia-ICO, Av. Granvia de L’Hospitalet 199-203, L’Hospitalet de Llobregat, 08908 Barcelona, Spain; Cancer Control and Prevention Group, Institut d’Investigació Biomèdica de Bellvitge-IDIBELL, Av. Granvia de L’Hospitalet 199-203, L’Hospitalet de Llobregat, 08908 Barcelona, Spain; Medicine and Health Sciences School, Universitat Internacional de Catalunya, C. Josep Trueta s/n, Sant Cugat del Valles, 08915 Barcelona, Spain; Biostatistic Unit, Department of Basic Sciences, School of Medicine and Health Sciences, Universitat Internacional de Catalunya, C. Josep Trueta s/n, Sant Cugat del Valles, 08915 Barcelona, Spain; Nursing Research Unit, Institut Català d’Oncologia-ICO, Av. Granvia de L’Hospitalet 199–203, L’Hospitalet de Llobregat (Barcelona), Spain, 08908 Barcelona, Spain; Addictions Unit, Institute of Neurosciences, Hospital Clínic de Barcelona - IDIBAPS, C. Villarroel 170, 08036 Barcelona, Spain; Department of Clinical Sciences, School of Medicine, Universitat de Barcelona, C. Feixa llarga s/n, L’Hospitalet del Llobregat, 08907 Barcelona, Spain; Department of Methodology for the Behavioural Sciences, University of Barcelona, Barcelona, Spain

**Keywords:** Hospital, Smoke-free policies, Health care providers, Smoking, Ban

## Abstract

**Background:**

Spain has passed two smoke-free laws in the last years. In 2005, the law banned smoking in indoor places, and in 2010 the ban was extended to outdoor areas of certain premises such as hospitals. This study assesses the impact of smoking consumption among hospital workers at a comprehensive cancer center after the passage of two national smoke-free laws.

**Methods:**

Six cross-sectional surveys were conducted among a representative sample of hospital workers at a comprehensive cancer center in Barcelona (2001–2012) using a standardized questionnaire. Logistic regression was used to compare differences in the odds of smoking after the laws took effect (baseline vs. 1^st^ law; 2^nd^ law vs. 1^st^ law).

**Results:**

Baseline smoking prevalence was 33.1%. After passage of the 1^st^ and 2^nd^ laws, prevalence decreased, respectively, to 30.5% and 22.2% (p for trend =0.005). Prevalence ratios (PR) indicated a significant decrease in overall smoking after the 2^nd^ law (PR = 0.65, 95% CI = 0.47-0-89). Smoking dropped in all professional groups, more prominently among those ≥35 years old, doctors, and women. Observed trends over the time included an increase in occasional smokers, a rise in abstinence during working hours but an increase in smoking dependence, and an increase in the employees’ overall support for the smoke-free hospital project.

**Conclusions:**

A long-term tobacco control project combined with two smoke-free national laws reduced smoking rates among health workers and increased their support for tobacco control policies. The decrease was more significant after the passage of the outdoor smoke-free ban.

## Background

Smoke-free policies are one of the most effective measures recommended by the World Health Organization Framework Convention on Tobacco Control (WHO FCTC) to control the tobacco epidemic [1, 2]. Smoking bans can be implemented by private organizations, accrediting agencies or boards, and local, state, or federal governments through legislation [3, 4]. Both governments and health organizations play leading active roles in tobacco control, sharing responsibility in providing primary healthcare, educating the community about tobacco-related issues, and assuring that public environments are healthy [5, 6].

Since the approval of the WHO FCTC, more than 120 countries [7, 8] and numerous health organizations have strengthened their smoke-free policies [9–12]. By means of these smoking bans, workers at hospitals that have either governmental or non-governmental smoke-free policies in place may benefit more than the general population from such policies. The benefits of smoke-free policies in health care services include: (a) decreased exposure to second-hand smoke (SHS) [13, 14]; (b) increased number of quit attempts and slightly reduced smoking consumption prevalence [15, 16]; and (c) a greater involvement in conducting tobacco prevention and cessation activities [17–20].

In 2005, Spain passed its first comprehensive smoke-free law, which banned smoking in indoor public places, workplaces, and health care services, except in hospitality venues [21]. In 2010, that law was amended providing: (1) smoke-free indoors for the hospitality sector (bars and restaurants) without exceptions, and (2) Smoke-free outdoors in some public areas including hospital grounds, educational campuses, and playgrounds [22]. However, some hospitals had already implemented their own regulations to prohibit smoking indoors before the first national law was enacted [23, 24]. Since 2002, the Catalan Institute of Oncology (ICO), a comprehensive cancer center in Barcelona, has developed several tobacco control policies by following the guidelines from the ENSH-Global Network for Tobacco-Free Health care Services (http://www.ensh.eu) [15, 25]. Throughout the 12 years of the hospital tobacco control project, several policies recommended in the ENSH model have been implemented, including: awareness campaigns on the hazards of SHS, smoke-free policies to protect people from SHS exposure, tobacco cessation services (including psychological support and pharmacotherapy if needed), and training courses for professionals, promotion activities, and evaluation efforts. These policies have been regularly monitored through diverse methods including smoking prevalence surveys, self-audit questionnaires, and observational inspections [10, 15, 24, 26, 27].

Although previous studies have monitored the psychosocial and behavioral effects of tobacco control policies at the hospital level [15, 28–30], those studies have not evaluated the impact of long-term institutional tobacco-free policies combined with the national smoke-free bans.

Given this context, the aim of this paper is to describe the impact of a 12-year tobacco control project (2000–2012) implemented in a comprehensive cancer center in combination with two national smoking bans (passed in 2005 and 2010). We describe trends in smoking consumption, attitudes, and behaviors among hospital workers at baseline (before passage of the 2 laws), after the 1^st^ law (which banned smoking indoors), and after the 2^nd^ law (which banned smoking on hospital grounds).

## Methods

### Design, procedure and sample

Six surveys were carried out among a representative sample of the employees of the Catalan Institute of Oncology from 2001 to 2012. According to the introduction of the national smoke-free bans, we defined the following evaluation periods: ***Baseline***, before the 1^st^ indoor smoke-free law: surveys in 2001, 2002 and 2004; ***after the 1***^***st***^***smoke-free law***, which banned smoking indoors: surveys in 2006 and 2009; ***after the 2***^***nd***^***smoke-free law***, which additionally banned smoking on hospital grounds: survey in 2012.

All surveys were conducted in the spring (from April to June) to avoid holiday seasons. Sample size estimation took into account the smoking prevalence among health care professionals in Catalonia, which was approximately 5 percentage points lower than prevalence in the general population [31]. The sample size was calculated using Statcalc in EpiInfo, version 6.0.4 (Centers for Disease Control and Prevention, Atlanta, US). Detailed sampling and data collection procedures have been previously reported [15]. However, in brief, a random sample of workers, based on age and sex group, was drawn from the Human Resources department updated files.

Research assistants located the index worker and provided him/her with the self-administered anonymous questionnaire contained in an envelope which the worker could use to return the questionnaire anonymously to maintain confidentiality. Participants absent from work on the interview days were contacted by the interviewers a maximum of four times at their work place. If subjects were not located, other subjects from the same group of age and sex were randomly selected as substitutes: substitution accounted for less than 12% of the corresponding sample in each of the six surveys.

### Questionnaire and variables

The survey instrument was developed by the experts’ working group from the ENSH-Global Network for Tobacco-free Hospitals (http://www.ensh.eu) [15] and adapted by the researchers. The questionnaires used in 2001 and 2002 were identical while the surveys used from 2004 to 2012 were shorter than the original version but maintained the following core variables: social and demographic data, profession, smoking status, and attitudes towards active and passive smoking.

The dependent variable was the prevalence of smoking. Subjects were classified according to smoking status as follows: daily smokers (currently smoking at least 1 cigarette/day), occasional smokers (currently smoking <1 cigarette/day), former smokers (not smoking for ≥6 months) [32], and never-smokers. We computed the prevalence (%) of smoking, including daily and occasional smokers. Among daily smokers, tobacco dependence was evaluated in terms of the number of cigarettes per day (<10, 10–20, and >20) and the time to the first cigarette after waking up (≤30 and >30 minutes). For all smokers, we collected additional information such as their concern about how smoking affected their own and others health, previous attempts to quit in the last year (yes, no), readiness to quit according to the stages of change model (pre-contemplation, contemplation, preparation) [33] -we considered “ready to quit” all responders in the contemplation and preparation stages-, readiness to set a date to quit at the moment of the interview (yes, no), previous consultation with a health professional to quit smoking (yes, no), and refraining from smoking in all areas of the hospital grounds (yes, no).

The main independent variables were sex, age, and profession, with the age variable divided into two separate categories (mean age <35 years or ≥35 years). Professional categories included *doctors, nurses, administrative employees,* and *other hospital workers* included mainly technicians, statisticians, researchers, and a very small number of workers who are phycologists (one in our organization), nutritionists (one in our organization). For some analyses, we categorized them as *health care providers* (nurses and doctors) and *non-health care providers* (administrative employees and others).

Finally, we surveyed all hospital workers to assess their support for the ‘Hospital Tobacco Control Project’, their agreement with the exemplary role that some groups should set (health care providers, teachers, and parents), and their opinion regarding the importance of raising taxes to effectively reduce tobacco consumption. Each of these questions had two response options (agree or disagree).

### Ethical considerations

Each survey administered was previously approved by the Institution Ethical Board of the Hospital Universitari de Bellvitge and participants gave oral consent to participate.

### Statistical analysis

The prevalence (%) of daily and occasional smokers, former smokers, and never-smokers of cigarettes, cigars or pipes, and 95% confidence intervals (CI) were computed. 99.6% of smokers were cigarette users. Smokers’ patterns of tobacco consumption were characterized in terms of tobacco dependence (number of cigarettes per day and time to first cigarette), readiness to quit, previous quit attempts, and previous consultation with a health professional to quit. To determine the trend over the time we computed the p-value for the linear trend for the target variables.

The impact of the two laws was assessed by fitting log-binominal regression models to obtain the prevalence ratio (PR) and 95% CIs for smoking after the 1^st^ law (surveys from 2006 and 2009) and after the 2^nd^ law (results from 2012) compared to baseline values (from surveys carried out in 2001, 2002, and 2004). We adjusted the models for sex, age, and profession, when necessary. All procedures were implemented using SPSS 18.0.

## Results

### Socio-demographic data

Approximately 200 workers were interviewed in each cross-sectional survey. Over the study period, the female-to-male ratio remained stable (75% females). However, the distribution of age changed, with the proportion of workers aged ≥35 years increasing during the study period. The professional status distribution also changed, with nurses accounting for 44.8% of participants at baseline to 34.9% after the passage of the 2^nd^ law; in contrast, representation of the “other professionals” increased from 21.4% at baseline to 26.2% after the 2^nd^ law (Table 1).

**Table 1:** **Demographic participants’ characteristics at baseline, after passage of the 1st and 2nd smoke-free laws**

	Baseline: 2001-2002-2004		After 1 ^st^ law: 2006-2009		After 2 ^nd^ law: 2012		p for trend
	(n = 580)		(n = 462)		(n = 221)		
	n	%	n	%	%	%	
***Sex***							
Men	150	25.9	111	24.0	52.0	23.5	0.429
Women	430	74.1	351	76.0	169.0	76.5	
***Age group (years)***							
18-24	28	4.8	25	5.5	7.0	3.2	<0.001
25-34	276	47.6	184	39.8	61.0	27.6	
35-44	191	32.9	173	37.4	95.0	43.0	
44-55	63	10.9	60	13.0	46.0	20.8	
> 55	22	3.8	20	4.3	12.0	5.4	
***Profession group***							
Doctors	104	17.9	81	17.5	41.0	18.6	0.060
Nurses	260	44.8	186	40.3	77.0	34.8	
Administrative employees	92	15.9	66	14.3	45.0	20.4	
Others	124	21.4	129	27.9	58.0	26.2	

### Smoking status

**Figure 1:**
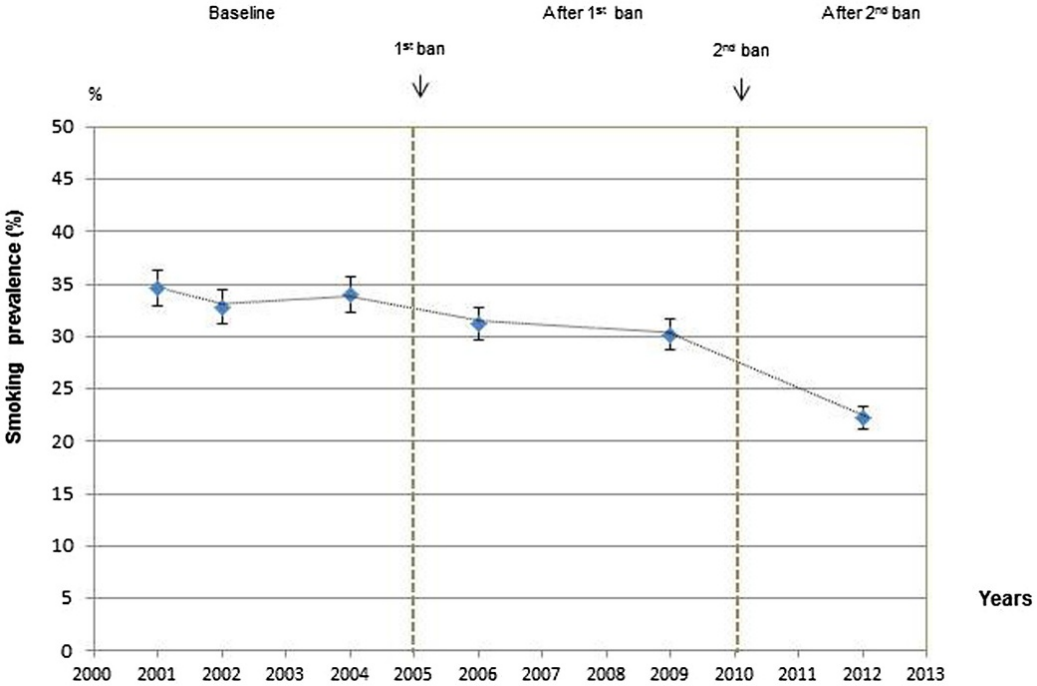
**Tobacco consumption prevalence and trends in the 3 periods: baseline, after 1** ^**st**^ **ban, after 2** ^**nd**^ **ban.**

At baseline, before passage of the national smoking laws, 33.1% (95% CI 29.3-36.9) of hospital workers were smokers; however, after the implementation of the 1^st^ smoke-free law –which banned smoking indoors- the prevalence decreased to 30.5% (95% CI 26.3-34.7), and after the implementation of the 2^nd^ law - which extended the smoking ban to outdoors- prevalence decreased to 22.2% (95% CI 16.7-27.6), with a statistically significant trend (p = 0.005; Table 2). After adjustment, the model confirms a lower prevalence of smokers after the 2^nd^ law (PR = 0.65, 95% CI: 0.47-0.89) (Table 2). Figure 1 shows the decline in tobacco consumption after the passage of the two laws.

**Table 2:** **Smoking status prevalence by selected independent variables at baseline, after passage of the 1** ^**st**^ **and 2** ^**nd**^ **smoke-free laws**

	Baseline 2001-2002-2004		After 1 ^st^ law 2006-2009		After 2 ^nd^ law 2012		p for trend
	(n = 580)		(n = 462)		(n = 221)		
	%	PR (Ref)	%	PR (95% CI) ^a^	%	PR (95% CI) ^a^	
**Never-smokers**	42.9	1	41.6	0.99 (0.82 - 1.20)	49.7	1.28 (1.01 - 1.62)	0.118
**Former smokers**	24.7	1	27.9	1.18 (0.99 - 1.46)	28.1	0.69 (0.82 - 1.50)	0.232
**Current smokers** ^**b**^	33.1	1	30.5	0.91 (0.73 - 1.13)	22.2	0.65 (0.47 - 0.89)	0.005
***Smoking prevalence by selected variables***							
***Sex***							
Men	27.3	1	22.5	0.77 (0.46 - 1.30)	19.2	0.59 (0.28 - 1.22)	0.200
Women	35.1	1	33.0	0.94 (0.74 - 1.21)	23.1	0.65 (0.46 - 0.94)	0.009
***Age group (years)***							
< 35	34.2	1	39.2	1.18 (0.87 - 1.58)	35.3	1.01 (0.64 - 1.59)	0.507
≥ 35	31.9	1	23.3	0.65 (0.47 - 0.91)	16.3	0.42 (0.27 - 0.67)	0.000
***Professional group***							
Doctors	22.1	1	17.3	0.72 (0.37 - 1.41)	15.0	0.20 (0.05 - 0.87)	0.018
Nurses	31.5	1	31.7	1.08 (0.77 - 1.52)	24.7	0.82 (0.49-1.38)	0.357
Administrative staff	41.3	1	27.3	0.61 (0.34 - 1.08)	33.3	0.78 (0.42 - 1.46)	0.222
Others	39.5	1	38.8	0.95 (0.66 - 1.47)	22.4	0.54 (0.29 - 1.01)	0.050

^a^Adjusted for sex, age, and profession when necessary.

^b^Includes daily and occasionally smokers.

PR: prevalence ratio obtained from a log-binomial regression model adjusted for sex, age group, and profession group when necessary.

By sex, the prevalence of smoking decreased progressively in both men and women over the three periods. Among men, smoking prevalence dropped from 27.3% at baseline to 19.2% after passage of the 2^nd^ law. Among women, smoking prevalence dropped from 35.1% at baseline to 23.1% after the 2^nd^ law, with an even greater reduction in prevalence after the 2^nd^ law compared to the 1^st^ law (Table 2). By age, workers ≥35 years old reduced their smoking prevalence after each of the two laws, with greater decrease observed after the 2^nd^ law (PR after 1^st^ law = 0.65, 95% CI: 0.47-0.91; PR after 2^nd^ law = 0.42, 95% CI: 0.27-0.67). Among the oldest workers (≥35 years old), the increased percentage of former smokers occurred mainly after the 1^st^ law (PR = 1.48, 95% CI: 1.09-2.02). The youngest workers (<35 years old) maintained a stable smoking consumption prevalence (~35%) before and after passage of the two smoke-free laws (Table 2).

Smoking prevalence decreased in all hospital worker groups during the study period. After the 2^nd^ law, doctors had the lowest prevalence (15.0%) while administrative staff had the highest (33.3%), with adjusted PRs of 0.20 (95%CI: 0.05-0.87) and 0.78 (95%CI: 0.42-1.46), respectively to baseline (Table 2).

### Smoking pattern

Table 3 shows the smoking pattern through the three evaluation periods. We observed that occasional smokers increased twofold, from 12.1% (95% CI: 7.5-16.7) at baseline to 24.5% (95% CI: 12.5-36.5) after passage of the 2^nd^ law.

**Table 3:** **Smoking pattern among smokers before law, after passage of the 1st and 2nd smoke-free**

	Baseline (n = 190)		After 1 ^st^ law (n = 138)		After 2 ^nd^ law (n = 49)		p for trend
	%	95% CI	%	95% CI	%	95% CI	
***Type of consumption***							
Daily smokers	87.90	(83.3-92.5)	79.0	(72.2-85.8)	75.5	(63.3-87.5)	0.012
Occasional smokers	12.10	(7.5-16.7)	21.0	(14.2-27.8)	24.5	(12.5-36.5)	
***Tobacco dependence***							
***Number of cigarettes per day***							
< 10 cigarettes	50.6	(43.5-57.7)	45.9	(37.5-54.2)	40.5	(26.7-54.2)	0.226
10-20 cigarettes	43.8	(36.7-50.8)	40.5	(32.3-48.7)	59.5	(45.7-73.2)	0.710
> 20 cigarettes	5.7	(2.4-8.9)	13.5	(7.8-19.2)	0.0	-	0.827
***First cigarette after awaking***							
≤ 30 minutes	3.6	(1.1-6.6)	6.0	(2.0-9.9)	39.1	(25.4-52.7)	<0.001
> 30 minutes	96.4	(93.7-99.0)	82.6	(76.3-88.9)	60.9	(47.2-74.5)	
***Willingness to quit***							
***Concerned about tobacco use effects***							
On their own health	69.7	(63.2-76.2)	71.7	(64.2-79.2)	62.5	(48.9-76.0)	0.594
On others health	66.0	(59.3-72.7)	46.2	(37.9-54.5)	48.9	(34.9-62.9)	0.002
***Previous quit attempts in the last year***							
Yes	57.4	(50.4-64.4)	56.5	(48.2-64.7)	72.3	(59.7-84.5)	0.163
***Readiness to quit***							
Yes	60.3	(56.1-69.8)	28.20	(20.8-35.7)	11.5	(2.2-19.7)	<0.001
***Readiness to fix a data to quit***							
Yes	36.5	(29.65-43.3)	31.40	(23.3-38.7)	20.4	(12.1-35.6)	0.036
***Consulted a professional to quit***							
Yes	10.1	(5.7-14.3)	40.7	(32.8-49.2)	65.3	(56.4-82.2)	<0.001
***Refrain from smoking in working hours***							
Yes	14.1	(9.15-19.0)	28.6	(21.1-36.1)	34.0	(20.7-47.3)	0.001

No clear trend was observed in the number of daily cigarettes and time to first cigarette, with oscillations during the three study periods. Nevertheless, after both smoke-free laws were passed, the percentage of smokers who smoked 10 to 20 cigarettes per day increased, and those that smoked their first cigarette ≤30 minutes after waking up increased from 3.6% at baseline to 39.1% after the 2^nd^ law (p for trend <0.001) (Table 3).

### Smokers’ concern about tobacco harmful effects

Overall, smokers’ concern about the health of others decreased from 66.0% at baseline to 48.9% after the second law (p for trend = 0.002). However, their concern for their own health remained stable over the 12-year period (Table 3).

### Attempts to quit smoking

At baseline, 57.4% of smokers reported having made a serious attempt to quit; after passage of the 2^nd^ law (Table 3), this percentage had increased to 72.3%. Over the study period the percentage of smokers who reported consulting a health professional to quit increased four-fold [from 10.1% to 40.7% to 65.3% respectively, at baseline, after the 1^st^ law, and after the 2^nd^ law; p for trend <0.001]. After the passage of the two smoke-free laws, the decrease in the percentage of smokers who considered themselves ready to quit was quite steep [from 60.3% at baseline to 11.5% after the 2^nd^ law (p for trend <0.001)]; similarly, the proportion of subjects expressing a desire to set a quit day also decreased substantially [from 36.5% at baseline to 20.4% after the 2^nd^ law (p for trend = 0.036)]. The percentage of smokers who refrain from smoking during working hours increased after each of the laws (from 14.1% at baseline to 34.0% after the 2^nd^ law, p for trend <0.001).

### Attitudes toward smoking and tobacco control policies

The majority of hospital workers agreed with the tobacco control policies implemented at the hospital, with a large increase in support following passage of the 2^nd^ law (p for trend <0.001) (Table 4). Nevertheless, when we compared health care providers (HCP) to non-HCP, we observed a significant trend only in the former group: 59% of HCP agreed with the tobacco control policies at baseline and 80.5% agreed with them after the 2^nd^ law (p = 0.000) (data not shown).

**Table 4:** **Agreement with the hospital tobacco control policy and some statements among all workers and smoker workers**

	Baseline		After 1 ^st^ ban		After 2 ^nd^ ban		p for trend
	(All n = 580/Smokers = 192)		(All n = 462/Smokers n = 141		(All n = 221/Smokers n = 49)		
	%	95% CI	%	95% CI	%	95% CI	
***Agreement with the hospital tobacco control policy***							
All	59.9	(55.9-63.8)	61.7	(65.2-73.8)	76.4	(70.8-81.9)	<0.001
Smokers*	57.8	(50.8-64.7)	53.2	(44.8-61.5)	71.4	(58.7-84.1)	0.320
***Health professionals should set an example and do not smoke***							
All	53.8	(49.7-57.8)	62.8	(58.4-67.2)	61.4	(72.2-83.2)	0.009
Smokers	43.8	(36.8-50.8)	47.5	(39.1-55.8)	22.4	(10.7-34.1)	0.068
***Teachers should set an example and do not smoke***							
All	56.2	(52.3-60.4)	62.6	(58.4-67.2)	64.5	(58.5-71.1)	0.014
Smokers	43.2	(36.2-50.2)	46.1	(37.8-54.4)	30.6	(17.7-43.5)	0.303
***Parents should set an example and do not smoke***							
All	68.8	(64.2-71.8)	70.8	(66.6-74.9)	77.3	(71.2-82.8)	0.027
Smokers	59.9	(52.9-66.8)	59.5	(51.3-67.7)	49.0	(35.0-62.9)	0.266
***Taxes should increase to decrease tobacco consumption***							
All	52.8	(48.4-56.6)	44.2	(65.2-73.8)	57.5	(50.89-64.0)	0.875
Smokers	37.0	(30.2-43.8)	39.0	(30.9-47.1)	36.7	(23.2-50.2)	0.892

*Smokers include daily and occasional smokers.

Agreement about the role of health professionals in setting a good example increased slightly among all workers over the study period (from 53.8% at baseline to 61.4% after the 2^nd^ law, p for trend = 0.009); however, after the 2^nd^ law, the agreement of smokers with this statement was 39 percentage points lower than the mean score of the whole group of workers (Table 4). HCP presented higher support (66.1%) to this statement after the 2^nd^ law if compared with non-HCP (33.9%, p < 0.05%)).

Finally, half of hospital workers agreed that taxes should be raised to effectively control tobacco consumption, without changes before and after implementation of the two smoke-free laws. Support for taxes among smokers was lower (37.0%) than the entire group of workers (52.8%; p < 0.05%) (Table 4).

## Discussion

The present study assesses, for the first time, the impact of a long-term tobacco control project in combination with two national smoke-free laws. Findings indicate a significant smoking reduction (mainly after passing the 2^nd^ law) and important changes in smoking patterns, including an increase in the proportion of occasional smokers, a rise in smoking abstinence during working hours, and an increase in support for the hospital tobacco control policies in the whole study population, particularly in non-smokers and health care providers. As previous studies indicate, the more restrictive the smoke-free policies, the greater the effects on smoking behavior [4, 34–36]. At baseline, smoking prevalence among hospital workers was similar to prevalence in the general population at that time (33.1% versus 32.1%, respectively) [37]. After the implementation of the 1^st^ law, prevalence remained similar to the general population (30.5% versus 29.4%, respectively) [31]; however, after implementation of the 2^nd^ law, smoking prevalence among hospital workers decreased sharply in comparison to the general population (22.2% versus 29.5%, respectively) [38]. This gradual decrease suggests an additive effect of the long-term organizational tobacco control policy in conjunction with national policies on hospital workers’ behavior.

Decreases in tobacco consumption were observed mainly in hospital workers ≥35 years old, doctors, and women. We hypothesize that the smoke-free legislation has had lower impact on the youngest group (<35 y) because young smokers tend to trivialize the harmful effects of smoking, and in our context there are insufficient initiatives addressed to motivate cessation among young smokers, even for health professionals”. It is also remarkable the decrease in smoking rates among women. Although, the hospital has not launched special campaigns addressed to them, we believe that the several cessation training programs addressed to nurses - who are 40% of our work force and from them 90% are women- may have had a stronger impact on quitting among this group. In addition, nurses smoking rates have had an appreciable decrease mainly after the passage of the 2^nd^ law.

Health workers are viewed as exemplary professionals by the rest of the society and thus they should be on the frontlines of tobacco control [39]. According to an international review that described smoking consumption among physicians, countries that implemented early tobacco control policies (e.g., the United States, Australia, and the United Kingdom) had a rapid decline in smoking prevalence among physicians, and currently those countries now have the lowest prevalence rates in the world [40]. A similar trend study conducted in Ireland showed also a striking decrease in smoking staff rates but with a stronger occupational gradient than in ours [41]. Our study reveals lower smoking rates among oncology nurses than among administrative and general population. However, oncology nurses at our institution still smoke more (by 10 percentage points) than oncology doctors, a finding that is consistent with the situation in other developed countries [42].

In the last decade, smoke-free policies in Europe have become more common in health care services due to the passage of both governmental [8] and non-governmental initiatives [43]. Nevertheless, despite the clear benefits of smoke-free policies [4], the WHO FCTC encourages organizations and governments to do more than just implement restrictions, advocating for the development of a broad tobacco control approach [1]. In this regard, our ‘Hospital Tobacco Control Project’ has developed a comprehensive tobacco-free model based on the ENSH-Global Network for Tobacco-free Health Care Services. The ENSH model integrates ten policies in agreement with Article 8, 12, 14, and 21 of the WHO FCTC (Article 8: “smoking bans in public places,” Article 12: “consumer information,” and Article 14: “access to treatment for quitting smoking,” Article 21: “research, surveillance and exchange of information”). The ENSH concept follows an organizational and cultural change model for implementing innovations [44] that has shown that a gradual implementation improves tobacco control policies [10, 45].

Many of the other policies recommended in the WHO FCTC have been poorly developed in health care services [46]. For instance, provision of tobacco cessation services (Article 14) is less than optimal [10, 47–49] and in many cases the programs offered form part of research studies, with a low likelihood of future sustainability [50, 51]. In our context, Catalan hospitals provide tobacco cessation services with the support of the regional government. Our comprehensive cancer center has offered tobacco cessation aid to workers since 2005, including behavioral support and free pharmacological treatment from 2005 to 2008. Afterwards, smoker workers should pay their own pharmacological treatment, and professional tobacco cessation consultation remains out of charge. A study assessing this intervention showed a high probability of abstinence at 6 months follow-up [26]. This result is in line with the substantial decrease in the prevalence of tobacco consumption among our hospital workers after the national bans, as well as the increase in the proportion of smokers who have consulted a health professional for help in quitting tobacco. In addition, the high dependence on cigarettes (i.e., the increase in subjects who have their first cigarette in ≤30 minutes of waking) and the low readiness to quit among our smoker workers is noteworthy, and seems to suggest that some “hardening” of smoking habits is occurring in this specific population [52]. Another remarkable point is the substantial increase in the number of occasional smokers in our hospital worker population. However, this finding is in line with other studies that have reported a similar increase in the number of the occasional smokers in countries where tobacco consumption in the overall population is decreasing [53] especially among some role model professions such as health careproviders [54].

Smoking cessation care in hospitals continues to present a challenge in many organizations [10, 47, 48]. Worldwide, the most commonly-identified barriers to smoking cessation efforts include: lack of resources, knowledge, time, and support [55–57]. The deficit in adequate tobacco cessation knowledge starts at the university level. According to a recent study, few health sciences degrees include tobacco cessation training in their curricula [58]. Also, constraints on financial and staffing resources may threaten the suitability of innovative projects [50, 51]. As a result neither health professionals nor hospital administrators see providing tobacco cessation services as part of their responsibilities [59]. In our study, hospital workers as a group increased their support for hospital tobacco control policies; however, agreement about the exemplary role of health professionals is still lower than desirable.

This study has limitations that must be considered when interpreting the results. First of all, our study was conducted at a comprehensive cancer center which has taken an active role in as a promoter of the tobacco control hospital model. Therefore, the remarkable decrease in tobacco consumption observed could be higher than in other hospitals. Nevertheless, in Catalonia, similar policies have been implemented at other public hospitals that are members of the Catalan Network for Smoke-free Hospitals (90% of public hospitals are members). This suggests that similar results can be expected at health care organizations/institutions that implement a long-term tobacco control policy that is supported by national smoke-free bans. Another potential limitation of our study is the possibility that cross-sectional surveys that use self-reported smoking status may suffer from information bias related to the increasing denormalization of tobacco consumption and associated attitudes to such consumption over time. However, data collected through cross-sectional surveys provide a real picture of the situation and this approach also prevents drop outs that typically occur during follow-up. It is well- known that hospitals have a high staff turnover rate, mainly among younger workers and the professional group “others”. The population in our sample was young and this was an important factor in determining the best design for our research aims. The inclusion of biological measures to confirm the accuracy of self-reported smoking would have improved the reliability of the data. However, previous studies have shown that self-reports are an adequate form of classifying smokers in observational studies [60].

## Conclusion

In conclusion, a long-term tobacco control strategy—which included sensitization campaigns, tobacco cessation training, cessation programs, and periodic monitoring and evaluation—in conjunction with two national smoke-free bans, helped to reduce smoking prevalence rates among hospital workers. However, convincing health care providers to become more involved in tobacco control is still a challenge. In addition to the array of tobacco control initiatives that organizations could undertake (such as education, cessation programs, and awareness campaigns), future actions to effectively decrease tobacco consumption and increase providers’ involvement will depend on the commitment of public health departments, agencies, and governmental bodies. These should encourage and support health care providers, and especially to nurses, their engagement in tobacco control in order that they become part of the solution of the tobacco epidemic.

## Abbreviations

CI: Confidence intervals
ENSH: ENSH-global network for tobacco-free health care services
ICO: Catalan institute of oncology
PR: Prevalence ratio
SHS: Second-hand smoke
WHO FCTC: World health organization framework convention on tobacco control.
